# Mistakes*Read before the Harvard Odontological Society, January 20, 1910.

**Published:** 1910-08-15

**Authors:** Charles T. Warner

**Affiliations:** Boston, Mass.


### MISTAKES.*

BY CHARLES T. WARNER, D.M.D., BOSTON, MASS.

One of our popular evening papers run a series of cartoons headed "Never Again," in which the hero or victim (the terms are often interchangeable) meets with various misadventures and always ends by raising his left hand and making the above declaration. All men are vain and King Solomon

*Read before the Harvard Odontological Society, January 20, 1910.

says, "All men are liars." We like to tell about our successes in public, but we dream about our failures when we are alone at night. From which do we learn the most? Speaking in the first person singular, I would say the failures and although my name is *not* George, I will do it and tell of a few.

When I put in my first gold filling as a student at school I would not believe the instructor when he told us that undercuts were necessary, especially as the patient said it hurt and objected strenuously. When she came in the next day with the filling in a paper, trouble in her eye, and told the whole room her opinion of me as a dentist in good West End English, I was glad it did hurt, was only sorry it did not hurt more, and quite agreed with the instructor that undercuts are desirable.

And so I submit this paper not primarily because I claim to be an expert on the subject, for I am far, far too modest for that, but chiefly for the discussion I hope it will produce.

After graduating, one bad blunder I made was in taking all my impressions for partial cases in composition, for the very simple reason that I did not know how, having never been instructed, to use plaster. To this day there are instructors at the school who will argue for this same practice. Thanks to the coaching of some of my good friends I am now able to get an accurate model to work from, and if there are any of you who do not know the pleasure that that implies, I advise you to try it and you will say with me "never again" for the composition.

A cause of chagrin within the last few years has been my inabitity to get the results that other men claim for two of the popular modern filling materials, gold inlays and silicate cements. Many men I have learned, by personal conversation and by their writing, have practically given up the old-fashioned plugged gold filling as being obsolete on acount of the excessive nervous strain to both the patient and operator.

An inlay has always seemed mechanically wrong except in certain adaptable cases where it could be properly seated and the force of mastication would not dislodge it, but tend to drive it more firmly to place. During the past summer it was my good fortune to have referred to me a lady from New York who was spending the summer at the North Shore, and who is the patient of a famous devotee of gold inlays. This lady brought in an inlay to be reset. A few days later she sent in a friend with the same trouble, and subsequently no less than six appeared, all with the same failing and all patients of the same man. Yet this man has publicly stated that he never had a gold inlay come out.

Am I the only person who occasionally puts in such a good looking silicate filling in a bicuspid or molar that the patient after looking in the glass, wants to know which tooth has been filled? And am I the only person who has that same filling come back in six months black as your hat, chipped at the edges, or perhaps broken in two? If I am not, you will probably say with me "never again" where the cavity includes the grinding surface.

One of the greatest sources of trouble to the dentist, I believe, is in the treatment of the pulp. there are three general ways of treatment, and examples of mistakes may be helpful.

First. Cocaine, which some men tell me is so successful that they use nothing else, and have thrown their arsenic bottle away. But what would they do in a case like this. A girl with an aching upper bicuspid. A large cavity extended to the pulp and when the exposure was touched with a broach she gave a realistic imitation of Wilbur Wright. Cocaine was used under pressure, and the pulp removed, when it was discovered that the girl was slightly mistaken in her sensation, for it was putrescent for half its length. She went home happy, but her happiness was short lived, for I found her waiting for me the next morning a wreck from a sleepless night, caused by pain. All remedies tried were useless until we appealed to the court of last resort—the forceps. The mistake? I had simply carried the infection through the end of the root with the cocaine and started an abscess.

Next we have arsenous acid, and that has its disadvantages as this case will show. A cavity extending to the pulp in the grinding surface of a molar and a mesial filling in the same tooth. I left the filling in place to aid in sealing the arsenic and told the man to come in if he had pain. He came. There was redecay at the cervical of the old filling, and the arsenic leaked out on the gum, causing a slough. Never again do I trust old fillings in similar cases.

Lastly, we have the system of mummification. The theory is very simple, for why try to remove the pulps from crooked canals when they can be tanned and made a better filling material than anything we can insert? A few years ago, like some of the rest of you, I bought a preparation, which for short we will call "Dr. Munyan's Magic Mummifier." It works on precisely the same principle usd by the old Egyptians, but I find it has this radical difference: that whereas the Egyptian mummie stay quietly and decently dead, the modern ones in the course of time will kick the cover off from their coffin and behave in a very indecorous manner.

There is another mistake to which we are all prone, and that is to the things we say to our patients, and especially, in that manner of the gentle art of wielding the hammer. How easy it is when a patient comes in with a mouth full of poor fillings to take a crack at Dr. Jones, who put them in—especially if we are not on any too friendly terms with Dr. Jones. But when we have tried to work for that self-same patient we are often obliged to mentally apologize to Dr. Jones and congratulate him on his great skill in being able to do anything for her.

Dr. Brackett has so well expressed himself in regard to this matter of our saying that I want to repeat it:

"The dentist should be very careful in all his expressions concerning others. Never let it be said that your office is a good place to get the news. Seldom introduce a personal topic of conversation, and then only to comment something which is good and worthy. Ascribe to others good intent and right motives; make allowances for mistakes, misunderstandings and misapprehension. Do not mind trifles. Cultivate a broad-mindedness and a high-mindedness. With the right spirit in us we shall find but most rarely in the work of any other practitioner anything to condemn or even to pass over in silence; but we shall find all along the way multitudes of instances for commendation.

Some of our patients come to us to have a tooth filled. Undivided diligence in filling the tooth is what is desired. Above all, do not burden others with any of your own cares or trials. Before you say anything question if saying it will serve any good and useful purpose or make anybody really wiser, better or happier. No one is benefitted by having you retail that you did not sleep last night, or that mosquitos have bitten you, or that you have dyspepsia, or that the cook has left and the plumber did not come. First, do not have troubles; second, when you have troubles, do not mind them; third, when you mind them, do not talk about them."

With equal earnestness I would say that we should be ever ready to listen to the troubles of others, and alert to be as helpful as we can to every one who is really in need of such cheer and comfort as we can give.

A lamentable case of inconsiderate cruelty of expression was under these circumstances. A skilled and careful dental practitioner had been nursing along for some time a case of disease of the mouth, which he recognized as malignant. The patient was very ill, but neither he nor any of his friends had knowledge of the grave significance of his malady. If I remember rightly, the patient's wife was of an hysterical temperament, and a victim of heart disease, which made anything of a mental shock a great risk to her. One day when the patient and his wife were in the office, the attending practitioner had a call from another dentist whom he politely asked to see the case. At the first glance the visitor blurted out, in a voice to be heard through consulting and waiting rooms: "Well, I don't know what you think about it, but I think it is a cancer." The drop had fallen, and the harm done by the inconsiderate expression could never be repaired.

To offset this I give you some more kindly instances. A gentleman, now past eighty years of age, has for a long time at annual examinations pointed out a bucal filling in a right lower second molar as having been made when he was twenty-six. Really, his present dentist refilled the cavity a few years ago, but he is quite content that the old-time service shall have the credit.

Nothing in the way of faith could be more implicit than one elderly lady's confidence in mutton tallow as a panacea for every sort of toothache. In her case the most intense odontalgic yields at once to free application upon the gum. Some of her friends have hard work to accept and use her remedy, but her dental advisor expresses only rejoicing that she has such a ready and effective resource.

In the beginning of my practice an esteemable old lady, long since of blessed memory, came in wearing a partial lower plate with two ordinary porcelain central incisors attached. She told me of her long-cherished and deep-seated repugnance to wearing artificial teeth, and of how, when her own teeth had dropped out, her dentist had mounted them on the plate, and she had such satisfaction in wearing them. He would have been a black-hearted wretch who could have destroyed the sweet illusion. You will note that the writer draws a line of distinction between wilfully deceiving and permitting others to hold opinions to which they are entitled. There opinion may be a mistake, but if they are happy in it, may it not be possible to make a mistake by correcting a mistake?—*The Journal.*

